# Economic advantage of outpatient shoulder arthroplasty: a Markov model analysis

**DOI:** 10.1016/j.jsea.2026.100029

**Published:** 2026-04-30

**Authors:** Akin A. Adio, Andrew Bouras, Diego Gonzalez-Morgado, Andrew Horn, Adam Lindsay, Joseph A. Abboud, Hafiz F. Kassam

**Affiliations:** a Perelman School of Medicine at the University of Pennsylvania, Philadelphia, PA, USA; b Nova Southeastern University, Kiran C. Patel College of Osteopathic Medicine, FL, USA; c The Hand & Upper Extremity Center of San Antonio, San Antonio, TX, USA; d Hoag Orthopaedics, Newport Beach, CA, USA; e St. Charles Center for Orthopedics and Neurosurgery, Bend, OR, USA; f Department of Orthopaedic Surgery, Rothman Orthopaedic Institute, Philadelphia, PA, USA; g Hoag Orthopaedic Institute, Newport Beach, CA, USA

**Keywords:** Markov, Outpatient, Total shoulder arthroplasty, Cost-effectiveness, Inpatient, Cost-utility

## Abstract

**Background:**

Outpatient total shoulder arthroplasty (TSA) has become increasingly common due to advances in perioperative care and policy changes. While prior studies have demonstrated lower short-term costs associated with outpatient TSA, its long-term economic value compared with inpatient TSA remains unclear.

**Methods:**

A Markov cost-utility model compared outpatient and inpatient TSA from a U.S. payer perspective over a 20-year horizon. Costs and quality-adjusted life-years (QALYs) were discounted at 3%. Cost-effectiveness was evaluated at a willingness-to-pay threshold of $100,000 per QALY, with probabilistic sensitivity analyses varying readmission and revision risks.

**Results:**

In the base case analysis, outpatient TSA was the dominant strategy. Mean discounted lifetime costs were lower for outpatient TSA compared with inpatient TSA ($23,375 vs. $28,402), resulting in a cost savings of $5,027 per patient. Outpatient TSA was associated with a modest increase in effectiveness, generating 10.436 QALYs compared with 10.427 QALYs for inpatient TSA (incremental gain, 0.009 QALYs). In probabilistic sensitivity analysis, outpatient TSA demonstrated a mean incremental cost of −$5,142 (95% uncertainty interval, −$13,642 to $3,109) and a mean incremental effectiveness of 0.009 QALYs (95% uncertainty interval, −0.034 to 0.057). At a willingness-to-pay threshold of $100,000 per QALY, outpatient TSA was cost-effective in 89.0% of simulations and economically dominant in 61.1% of simulations.

**Conclusions:**

Outpatient TSA is a cost-effective and economically dominant strategy compared with inpatient TSA, driven primarily by lower initial procedural costs. Sensitivity analyses demonstrated that outpatient TSA remained cost-effective even when differences in perioperative complication and revision rates were substantially reduced.

Outpatient total shoulder arthroplasty (TSA), including anatomic and reverse total shoulder arthroplasty, has become increasingly common with advances in perioperative care and anesthesia techniques.^19^^,^^26^ Compared with total hip and knee arthroplasty, TSA may be associated with lower complication rates, reduced costs, and shorter hospital length of stay.^10^ Patients undergoing TSA are increasingly younger, which has driven growing interest in outpatient and same-day discharge pathways.^4^^,^^23^ Policy changes, including the removal of TSA from the Centers for Medicare and Medicaid Services (CMS) “inpatient-only” list, have further accelerated adoption of outpatient TSA.^22^

Beyond lower initial procedural costs, outpatient TSA is associated with lower resource utilization and a more favorable short-term cost profile compared with inpatient surgery.^24^ These differences are driven largely by shorter hospital stays and decreased rates of early post-operative events that increase episode-of-care costs.^16^ Prior studies have examined cost differences and short-term outcomes between inpatient and outpatient TSA using retrospective cohort designs and administrative databases.^12^^,^^14^ However, no prior study has evaluated the long-term economic value of outpatient vs. inpatient TSA using a decision-analytic Markov modeling framework that accounts for revision risk, readmissions, and lifetime outcomes.

Given the rapid adoption of outpatient TSA, its long-term economic value relative to inpatient care remains unclear. The purpose of this study was to evaluate the cost-effectiveness of outpatient vs. inpatient TSA using a decision-analytic Markov model. We hypothesized that outpatient TSA would be cost-effective compared with inpatient TSA at commonly accepted willingness-to-pay thresholds.

## Methods

### Overview of Markov modeling

Markov state-transition models are widely used in health economic analyses to simulate chronic disease processes and long-term treatment outcomes through transitions between a series of discrete health states.^29^ They simulate patients moving between defined health states at set intervals, with each state assigned a cost and quality-of-life value. Transitions between states are governed by probabilities that represent the chance of clinical events occurring during each cycle.^12^ These probabilities are derived from published literature, registry data, or expert consensus when high-quality evidence is unavailable. Quality-of-life is measured on a scale ranging from 0, representing death, to 1, representing perfect health, and is commonly expressed using validated instruments such as the European Quality of Life 5-Dimensions.^25^ During each cycle, patients accrue quality-adjusted life-years (QALYs) and costs based on the state they occupy. Over the lifetime horizon of the model, cumulative QALYs and costs are calculated for each treatment strategy. The principal outcome of a Markov cost-utility analysis is the incremental cost-effectiveness ratio, defined as the difference in cost between 2 strategies divided by the difference in QALYs gained.

In this analysis, the strategies compared were outpatient TSA and inpatient TSA. Cost-effectiveness was evaluated using a willingness-to-pay threshold of 100,000 US dollars per QALY, reflecting commonly accepted societal benchmarks.^20^ Strategies with an incremental cost-effectiveness ratio below this threshold were considered cost-effective, whereas strategies associated with lower costs and greater health benefits were considered dominant.

### Model structure

This study was conducted and reported in accordance with the Consolidated Health Economic Evaluation Reporting Standards guidelines.^15^ A state-transition Markov model was developed to evaluate the long-term costs and health outcomes associated with outpatient vs. inpatient TSA, including anatomic and reverse total shoulder arthroplasty. The model did not differentiate between anatomic and reverse total shoulder arthroplasty. Simplified pathways were used to represent complications and revisions to maintain model parsimony and because of limited high-quality data differentiating these outcomes across settings and implant types. Time in the model was divided into discrete monthly cycles to allow accurate representation of short-term perioperative events without overstating their duration or quality-of-life impact. The model was evaluated over a 20-year lifetime horizon to capture long-term revision risk and survival following primary TSA. Baseline starting ages of 66 and 70 years were used for outpatient and inpatient cohorts, respectively, based on Daher et al,^8^ with sensitivity analyses performed varying age by ±5 years to account for differences in patient populations. Costs and health outcomes were discounted at an annual rate of 3 percent in accordance with established health economic guidelines.

**Figure 1:**
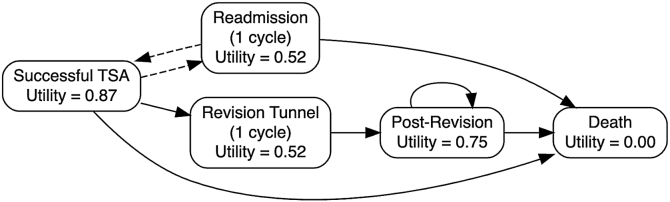
Markov state-transition model for outpatient and inpatient total shoulder arthroplasty. *TSA*, total shoulder arthroplasty.

The model consisted of 4 mutually exclusive health states: (1) successful primary TSA, representing the post-operative “well” state; (2) readmission; (3) postrevision; and (4) death, modeled as an absorbing state using age-adjusted mortality from 2021 United States Life Tables. An absorbing state is a health state that, once entered, cannot be left. The transition probability out of that state is zero. Post-operative complication and readmission risks were modeled over a 90-day post-operative period, consistent with standard arthroplasty outcomes reporting. Revision and readmission risks were applied conditional on survival, with death treated as a competing absorbing state. An overview of the model structure is presented in Figure 1.

### Data sources

This study was a decision-analytic Markov model using data derived from previously published literature and publicly available sources. As no patient-level data were collected or analyzed, this study did not constitute human subjects research and was therefore exempt from institutional review board approval. All inputs, ranges, and sources are stated in Table I.

**Table I:** Model inputs and health state utilities used in the Markov analysis.

Parameter	Outpatient	Inpatient	Ranges	Source
Age				
Age	66	70	61-71 (OP); 65-75 (IP)	Daher et al.
Costs				
Primary surgery	$16,941	$20,584	$13,553-$20,329 (OP); $16,467-$24,701 (IP)	Agarwal et al.
Readmission	$4,237	$4,237	$3,390-$5,084	Corso et al.
Revision surgery	$46,942	$46,942	$33,588-$50,382	Corso et al.
Probabilities				
Readmission	3.60%	5.20%	2.8%-4.6% (OP); 4.6%-6.9% (IP)	Al-Sadawwi et al.
Revision risk	3.60%	5.10%	2.3%-3.8% (OP); 3.8%-5.7% (IP)	Al-Sadawwi et al.
Utilities				
Successful TSA (well)	0.87	0.87	0.78-0.96	Grobet et al.
Postrevision state	0.75	0.75	0.68-0.83	Davies et al.
Temporary readmission state	0.52	0.52	0.46-0.60	Coe et al.
Temporary perioperative revision state	0.52	0.52	0.46-0.60	Coe et al.

Parameter	Outpatient	Inpatient	Source	Source
Costs				
Primary surgery	$16,941	$20,584	$13,553-$20,329 (OP); $16,467-$24,701 (IP)	Agarwal et al.
Readmission	$4,237	$4,237	$3,390-$5,084	Corso et al.
Revision surgery	$46,942	$46,942	$33,588-$50,382	Corso et al.
Probabilities				
Readmission	3.60%	5.20%	2.8%-4.6% (OP); 4.6%-6.9% (IP)	Al-Sadawwi et al.
Revision risk	3.60%	5.10%	2.3%-3.8% (OP); 3.8%-5.7% (IP)	Al-Sadawwi et al.
Utilities				
Successful TSA (well)	0.87	0.87	0.78-0.96	Grobet et al.
Postrevision state	0.75	0.75	0.68-0.83	Davies et al.
Temporary readmission state	0.52	0.52	0.46-0.60	Coe et al.
Temporary perioperative revision state	0.52	0.52	0.46-0.60	Coe et al.

*OP*, outpatient; *IP*, inpatient; *TSA*, total shoulder arthroplasty.

### Clinical inputs and transition probabilities

Revision and readmission risk estimates were derived from a recent meta-analysis by Al-Sadawwi et al,^3^ which included 428,127 patients across 34 studies evaluating outcomes following outpatient and inpatient TSA. Revision rates for outpatient TSA was reported to be 3.61%, whereas the revision rate for inpatient TSA was 5.10%. These estimates found to be consistent with long-term revision patterns reported by the Australian Orthopedic Association National Joint Replacement Registry.^17^ Hospital readmission rates for the outpatient and inpatient cohorts in that registry were 3.64% and 5.20%, respectively.^3^ If a readmission occurred, it resulted in a single additional cost and a short-term reduction in quality of life, with no ongoing effects in later cycles. Perioperative mortality was low (<1%) and comparable between outpatient and inpatient TSA, as confirmed by multiple prior studies.^11^^,^^18^

### Costs

The analysis was conducted from a United States payer perspective, with all costs expressed in 2024 US. dollars. The base procedural cost for outpatient TSA was $16,941, compared with $20,584 for inpatient TSA, derived from a national database.^1^ Complications requiring hospital readmission incurred a weighted cost of $4,237, while revision surgery was assigned a comprehensive episode cost of $46,942.^7^ Future costs were discounted at an annual rate of 3.0%.^27^

### Health state utilities

Health state utilities were derived from published European Quality of Life 5-Dimensions–based studies. Patients in the Successful TSA state were assigned a utility of 0.87.^13^^,^^14^ Readmission and acute revision tunnel states were assigned a utility of 0.52 for a single monthly cycle.^6^ Patients transitioning to the postrevision state were assigned a stable utility of 0.75.^9^ Death was modeled as a utility of 0.00. Future utilities were discounted at 3.0% annually.^29^

### Sensitivity analysis

All Markov modeling and probabilistic sensitivity analyses were performed using TreeAge Pro (TreeAge Software, Williamstown, MA, USA). Probabilistic sensitivity analysis was performed using Monte Carlo simulation with 5,000 iterations. Beta distributions were assigned to transition probabilities and utilities, and Gamma distributions to cost parameters. Cost-effectiveness was assessed using Incremental Net Monetary Benefit (INMB) at a willingness-to-pay threshold of $100,000 per QALY.

## Results

### Base-case analysis

In the deterministic base case analysis, outpatient TSA was the dominant strategy. Mean discounted lifetime costs were lower for outpatient TSA ($23,375) compared with inpatient TSA ($28,402), yielding a cost savings of $5,027 per patient (Table II). Outpatient TSA was also associated with a modest increase in effectiveness, generating 10.436 QALYs versus 10.427 QALYs for inpatient TSA, corresponding to an incremental gain of 0.009 QALYs.

**Table II:** Base case cost and effectiveness outcomes for outpatient vs. inpatient total shoulder arthroplasty.

Outcome	Outpatient	Inpatient	Difference
Total Ccost	$23,375	$28,402	-$5,027 (savings)
Total QALYs	10.436	10.427	+0.009 (gain)

*QALY*, quality-adjusted life-year.

### Probabilistic sensitivity analysis

**Figure 2:**
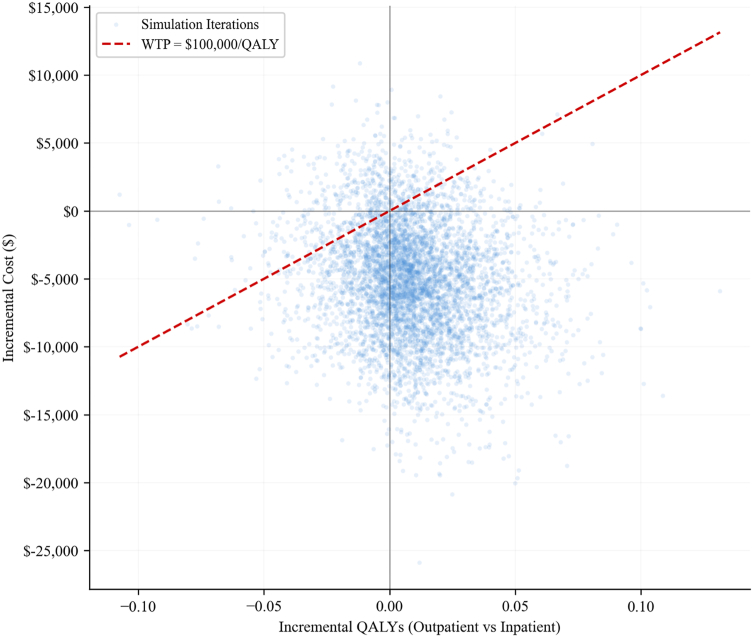
Cost-effectiveness plane from probabilistic sensitivity analysis (5,000 Monte Carlo iterations). Each point represents one simulation. The dashed line represents the WTP threshold of $100,000 per quality-adjusted life-year. *QALY*, quality-adjusted life-year; *WTP*, willingness-to-pay.

Probabilistic sensitivity analysis demonstrated the robustness of these findings. The majority of simulations clustered in the southeast quadrant of the cost-effectiveness plane, indicating lower costs and higher effectiveness for outpatient TSA (Fig. 2). Outpatient TSA was associated with a mean incremental cost of −$5,142 (95% uncertainty interval: −$13,642 to $3,109) and a mean incremental gain of 0.009 QALYs (95% uncertainty interval: −0.034 to 0.057). At a willingness-to-pay threshold of $100,000 per QALY, outpatient TSA was cost-effective in 89.0% of simulations and dominant in 61.1% of simulations.

### One-way deterministic sensitivity analysis

**Figure 3:**
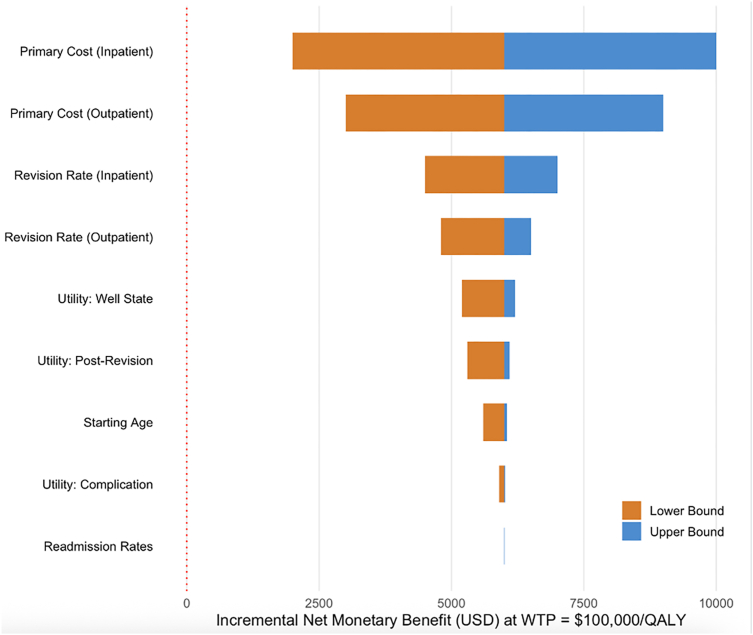
One-way deterministic sensitivity analysis (tornado diagram) showing the influence of individual parameter variation on incremental net monetary benefit at a WTP threshold of $100,000 per quality-adjusted life-year. Primary procedural costs were the most influential parameters. *QALY*, quality-adjusted life-year; *WTP*, willingness-to-pay.

One-way deterministic sensitivity analysis demonstrated that the cost-effectiveness of outpatient TSA was most sensitive to primary procedural costs (Fig. 3). Varying inpatient primary cost across its plausible range produced the largest swing in INMB ($8,234), followed by outpatient primary cost ($6,776) and inpatient revision rate ($2,945). Outpatient TSA remained cost-effective across the full range of all parameters tested, with INMB remaining positive in every scenario. Utility parameters and readmission costs had comparatively modest influence on the results.

## Discussion

This cost-utility analysis, using the most current available evidence on outpatient and inpatient TSA, demonstrates that outpatient TSA is a cost-effective strategy. In the base-case scenario, outpatient TSA was associated with lower lifetime costs and slightly higher QALYs compared with inpatient surgery, resulting in economic benefit. The incremental QALY gain observed in this study was modest and may not represent a clinically meaningful difference between outpatient and inpatient TSA. Rather, the economic advantage of outpatient TSA is primarily driven by lower procedural costs, with comparable effectiveness between strategies. This finding supports the interpretation that outpatient TSA offers similar clinical outcomes at reduced cost, rather than a substantial improvement in patient health status.

Multiple studies have consistently shown that performing TSA in the outpatient setting is associated with substantially lower costs compared with inpatient care.^1^ A Medicare claims analysis of over 100,000 matched TSA/rTSA cases found that outpatient surgery was associated with approximately 20% lower 90-day total costs compared with inpatient procedures.^5^ Yian et al^30^ demonstrated that outpatient shoulder arthroplasty can be 22% less expensive than inpatient TSA, with savings driven by reduced facility fees, hospital bed charges, and shorter overall episodes of care. Similarly, Ode et al^21^ further showed that ambulatory surgery center charges for shoulder arthroplasty are roughly 40% lower than combined inpatient charges even after adjustment for demographics and comorbidity differences. Though the magnitude of savings varies by methodology and payer perspective, the overall economic literature supports a consistent cost advantage for outpatient TSA in appropriately selected patients. The findings from the present model, specifically the deterministic base-case analysis support these cost advantages.

Although many studies report that outpatient TSA is associated with favorable outcomes, these findings are heavily influenced by patient selection practices. In a meta-analysis of 2,400 patients, Daher et al^8^ reported significantly younger patients and lower American Society of Anesthesiologists classifications in outpatient cohorts compared to inpatient cohorts. Another meta-analysis revealed no significant differences once baseline comorbidity burden is controlled in subgroup analyses limited to matched or adjusted cohorts.^2^ This suggests that differences in outcomes may reflect underlying health status rather than care setting alone. Overall, existing literature remains largely retrospective, with a true causal inference between setting and long-term outcomes remaining unresolved. To address this uncertainty, we tested variability in costly downstream events after TSA, including readmission and revision. Even when revision and readmission rates differed by as little as 0.1%, outpatient TSA remained more cost-effective, driven primarily by its lower initial procedural cost. These findings suggest that procedural cost differences play a larger role in determining value than small differences in complication incidence in appropriately selected patients.

In addition, the use of simplified complication and revision pathways reflects a limitation in the current literature. Most studies comparing outpatient and inpatient TSA report aggregate complication and revision rates rather than detailed clinical trajectories, limiting the ability to model timing, severity, or downstream events.^3^ For example, while revision rates are generally reported as similar between settings, they are not stratified by early vs. late failure or staged reoperations, which could meaningfully alter cost and QALY estimates. Incorporating these distinctions could improve model precision, particularly given differences in readmission and utilization patterns across settings. Currently, this would require assumptions not supported by current data. Future studies with longitudinal, procedure-specific complication pathways will be necessary to enable more granular and clinically representative modeling.

These findings have important implications in the context of evolving CMS policy. The removal of TSA from the Medicare Inpatient-Only list in 2021 eliminated a key structural barrier to outpatient reimbursement, enabling rapid migration of cases to ambulatory settings. This shift has already been associated with a marked increase in outpatient TSA volume without a corresponding increase in short-term complications or readmissions.^28^ Our results extend these findings by demonstrating that this transition is not only clinically feasible but also economically advantageous over the long term. From a policy perspective, this supports continued CMS efforts toward value-based care and bundled payment models, in which total episode cost is prioritized. Importantly, as reimbursement differentials between inpatient and outpatient settings persist, these findings may incentivize both providers and payers to preferentially shift appropriate cases to outpatient environments. In addition, alignment of financial incentives across health systems is important as outpatient migration may reduce inpatient revenue streams and alter traditional hospital cost structures.

### Limitations

This study has limitations inherent to Markov-based economic modeling. The model simplifies clinical care into a limited number of pathways and reflects an average patient experience rather than all possible perioperative courses. The analysis assumes that patients undergoing outpatient TSA are eligible for ambulatory surgery centers. As a result, findings should not be generalized to higher-risk populations who require inpatient care. In addition, while key parameters were varied across wide ranges in sensitivity analyses, unmeasured confounding in the underlying observational data may still influence estimates of costs and outcomes. For example, this analysis was conducted from a standardized U.S. payer perspective and may not capture variability across alternative reimbursement models, including cost-plus, bundled payment, or commercial pricing structures. In addition, differences in implant selection and cost structures between anatomic and reverse shoulder arthroplasty were not explicitly modeled and may influence economic outcomes. Moreover, the model did not include a separate re-revision state. Although clinically relevant, limited comparative data between outpatient and inpatient TSA would require additional assumptions that may reduce model validity. Lastly, this analysis did not differentiate between anatomic and reverse total shoulder arthroplasty, including augmented anatomic and augmented reverse total shoulder arthroplasty. Implant-specific costs and revision patterns were therefore not explicitly modeled, although prior data suggest similar inpatient vs. outpatient cost trends across implant types.^30^ Despite these limitations, the direction and magnitude of the findings were consistent across analyses and prior literature, supporting the validity of the results.

## Conclusion

In this cost-utility analysis, outpatient TSA was associated with lower lifetime costs and comparable long-term health outcomes compared with inpatient surgery, due in large part to the overall initial cost of care. The cost advantage persisted across extensive sensitivity analyses and was driven primarily by lower initial procedural costs rather than small differences in downstream events. These findings support the economic value of outpatient TSA in appropriately selected patients. Future studies should focus on refining patient selection criteria and evaluating outcomes in broader populations as outpatient TSA continues to expand.

## Disclaimers:

Funding: No funding was disclosed by the authors.

Conflicts of interest: Joseph Abboud would like to disclose: Royalties from a company or supplier; Disclosures; OSTEOCENTRIC TECHNOLOGIES, ENOVIS, ZIMMER-BIOMET, STRYKER, GLOBUS MEDICAL, INC. Stocks in: SHOULDER JAM, AEVUMED, OBERD, OTS MEDICAL, ORTHOBULLETS, ATREON, RESTORE 3D. Research support from a company or supplier as a PI; Disclosures; ENOVIS, ARTHREX. Royalties, financial or material support from publishers; Disclosures; WOLTERS KLUWER, SLACK ORTHOPAEDICS, ELSEVIER. Board member/committee appointments for a society; Disclosures; AMERICAN SHOULDER AND ELBOW SOCIETY, MID ATLANTIC SHOULDER AND ELBOW SOCIETY, SHOULDER 360, PACIRA.

Hafiz Kassam would like to disclose: Consultant for Zimmer Biomet and Smith & Nephew.

Any additional authors, their immediate families, and any research foundations with which they are affiliated have not received any financial payments or other benefits from any commercial entity related to the subject of this article.
